# Cyclodextrin–polysaccharide-based, in situ-gelled system for ocular antifungal delivery

**DOI:** 10.3762/bjoc.10.308

**Published:** 2014-12-08

**Authors:** Anxo Fernández-Ferreiro, Noelia Fernández Bargiela, María Santiago Varela, Maria Gil Martínez, Maria Pardo, Antonio Piñeiro Ces, José Blanco Méndez, Miguel González Barcia, Maria Jesus Lamas, FranciscoJ Otero-Espinar

**Affiliations:** 1 Pharmacy and Pharmaceutical Technology Department, Faculty of Pharmacy, University of Santiago de Compostela (USC), Praza Seminario de Estudos Galegos s/n, Santiago de Compostela, 1570, Spain; 2 Pharmacy Department, Xerencia de Xestión Integrada de Santiago de Compostela, SERGAS, Travesía Choupana s/n, Santiago de Compostela, 15706, Spain; 3 Ophthalmology Department, Hospital de Conxo, Xerencia de Xestión Integrada de Santiago de Compostela, SERGAS, Rua Ramón Baltar s/n, Santiago de Compostela, 15706, Spain; 4 Grupo Obesidomica, Instituto de Investigación Sanitaria (IDIS-ISCIII), SERGAS, Travesía da Choupana s/n, Santiago de Compostela, 15706, Spain; 5 Industrial Pharmacy Institute, Faculty of Pharmacy, University of Santiago de Compostela (USC), Praza Seminario de Estudos Galegos s/n, Santiago de Compostela, 15701, Spain

**Keywords:** cyclodextrins, eye drops, fluconazole, hydroxypropyl-β-cyclodextrin, sulfobutylether-β-cyclodextrin

## Abstract

Fluconazole was studied with two different hydrophilic cyclodextrins (hydroxypropyl-β-cyclodextrin (HPBCD) and sulfobutyl ether-β-cyclodextrin (SBECD)) for the formation of inclusion complexes. HPBCD and SBECD showed low cell cytotoxicity in human keratocytes as assessed by the label-free xCELLigence system for real-time monitoring. The fluconazole–HPBCD complex was incorporated into an ion-sensitive ophthalmic gel composed of the natural polysaccharides gellan gum and κ-carrageenan. This system showed good bioadhesive properties and effective control of fluconazole release.

## Introduction

Fungal keratitis is a serious disease that can lead to loss of vision. *Candida albicans* is one of the most widespread fungal pathogens involved in fungal keratitis [1–2]. The most common antifungal treatments include the use of polyenes and azoles; however, a significant treatment failure rate exists with these pharmacological agents [3]. Specifically, azole-based medications, which include imidazoles (e.g., miconazole, clotrimazole, and ketoconazole) and triazoles (e.g., fluconazole, itraconazole, posaconazole, and voriconazole), inhibit ergosterol synthesis in the cell wall [4].

Fluconazole is considered to be a good therapeutic option for most eye fungal infections; however, there are resistant species that require higher concentrations than usual [5]. Unfortunately, antifungal eye drops have low ocular bioavailability due to their poor water solubility and known effective eye clearance mechanisms [6]. Thus, after administration of volumes in the range of 25–50 µL, a rapid drainage occurs from the front of the eye. Additionally, due to the low volume that the eye can accommodate, an overflowing of the solution takes place. In most commercial formulations, the administered volume is about 30 µL, which is the mean volume of the human conjunctiva sac. However, it has been shown that after a single blink, only 10 µL of the solution remains [7].

In this context, pharmaceutical researchers have paid special attention to the development of ophthalmic drug delivery systems during the last decade. Different strategies have been studied to prolong the ocular surface–drug permanence time, to increase drug corneal permeability and also to delay drug elimination [8–9]. Hence, the in situ formation of polymer-based delivery systems has received increased attention due to their manageability and prolonged ocular residence time, which improves patient compliance and comfort [10]. Specifically, stimuli-responsive polymer hydrogels have recently attracted special attention as drug delivery systems due to their capacity to respond to changing environmental conditions such as ion composition, pH, temperature, presence of chemicals, or changes on electrical field, which result in the modification of its structure and properties [11]. Gellan gum (GG) and carrageenans are polysaccharides that can be used to enhance ion-sensitive hydrogels. Gellan gum is a linear anionic heteropolysaccharide with high molecular mass formed by a tetrasaccharide repeating unit of glucose, glucuronic acid and rhamnose in the ratio 2:1:1. Similarly, κ-carrageenan (CK) is also an anionic heteropolysaccharide with high molecular mass formed by repeating units of sulfated and nonsulfated galactose and anhydrogalactose joined by β-1→4 and α-1→3 glycosidic linkages. Aqueous solutions of both polysaccharides have characteristic properties related to their temperature dependence and cation-induced gelation [12].

A challenge in the design of bioadhesive ion-sensitive hydrogels is the incorporation of drugs with poor aqueous solubility. Cyclodextrins (CDs) are useful pharmaceutical excipients that aid in the formulation of poorly aqueous soluble drugs in a wide range of delivery devices from the classical dosage forms to the newest drug carriers [13]. Their spatial structure together with the great variety of substituents can provide versatile pharmaceutical advantages. 2-Hydroxypropyl-β-cyclodextrin (HPBCD) and sulfobutyl ether-β-cyclodextrin (SBECD) are two chemically modified cyclodextrins frequently used as vehicles to improve drug solubility. The aim of this work is to obtain an ophthalmic drug delivery system for the release of fluconazole (FC) based on the use of ion-sensitive bioadhesive hydrogels.

## Results and Discussion

### Phase solubility diagrams

**Figure 1:**
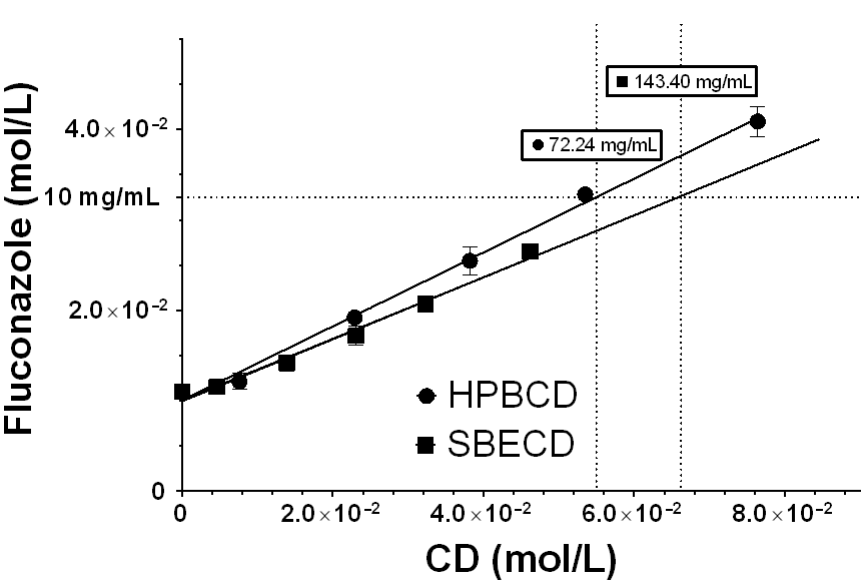
Phase solubility diagrams for fluconazole obtained with β-cyclodextrin derivatives at 25 °C in water (mean ± SD, *n* = 6). The dotted lines indicate the necessary CD concentration required to solubilize 10 mg/mL of fluconazole.

Phase solubility diagrams for fluconazole with HPBCD and SBECD at 25 °C are shown in Figure 1. With both cyclodextrins, A_L_-type curve diagrams were obtained [14], which suggests the formation of highly soluble inclusion complexes. Solubility curves can assess drug–cyclodextrin interactions that include the formation of inclusion and non-inclusion complexes. Additionally, they are useful to determine which cyclodextrin host, HPBCD or SBECD, was interacting more successfully with fluconazole [15]. Apparent stability constants (*K*_1:1_), complexation efficiency (CE) and the D:CD ratio for each cyclodextrin were calculated and values are listed in Table 1.

**Table 1:** Values for *K*_1:1_*,* CE and the D:CD ratio obtained from the fluconazole–cyclodextrin complex in water at 25 °C.

	S_0_ (M)	S_0 extrap_ (M)	*K*_1:1_ (M^−1^)^a^	*K*_1:1 extrap_ (M^−1^)^b^	CE	D:CD (mol:mol)	R^2^

HPBCD	1.1 × 10^−2^	1.0 × 10^−2^	62.73	69.00	0.69	1:2.45	0.9897
SBECD	1.1 × 10^−2^	1.0 × 10^−2^	46.81	51.49	0.51	1:2.96	0.9789

^a^*K*_1:1_ calculated using S_0_ (solubility of free drug); ^b^*K*_1:1_ calculated using S_0,extrap_ (free drug solubility calculated from the phase solubility diagram).

*K*_1:1_ values for the complexes obtained with the two β-CD derivatives were similar to those calculated for the parent β-CD by Upadhyay et al. [16] and for HPBCD by Kutyła et al. [17], evaluated using NMR spectroscopy. These authors describe an inclusion complex in water with a *K*_1:1_ value of 68.7 M^−1^ for the β-CD:fluconazole complex and 34.6 M^−1^ for the HPBCD:fluconazole complex; thus, they propose, the insertion of the *m*-difluorophenyl ring of the drug into the wide end of the torus cavity of both CDs. This geometry is likely compatible with the SBECD complex and therefore, no differences between both complexes can be expected regarding the *K*_1:1_, CE and D:CD ratio values.

Due to the high molecular weight of SBECD compared to HPBCD, the quantity of SBECD necessary to solubilize the fluconazole is higher than HPBCD. Thus, to solubilize a concentration of fluconazole of 10 mg/mL, a concentration of HPBCD of 72.24 mg/mL versus 143.40 mg/mL of SBECD (Figure 1) is necessary.

**Figure 2:**
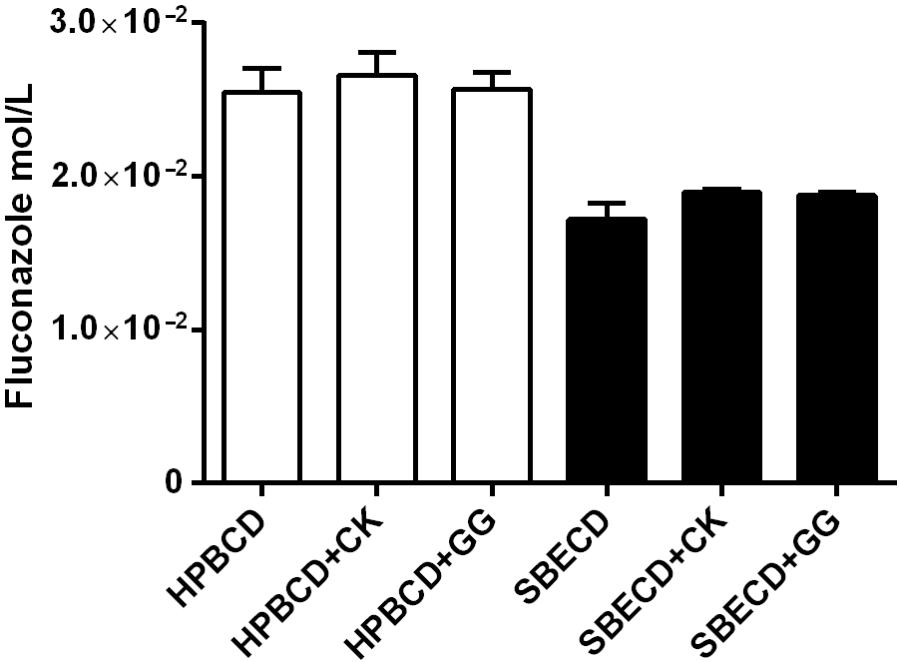
Solubility of fluconazole in aqueous solutions containing 5% of CD and 0.5% of the respective polysaccharide.

One of the main purposes of this study was to design a bioadhesive hydrogel using ion-sensitive natural polymers. To achieve this goal, two natural polysaccharides, gellan gum and κ-carrageenan were incorporated into the formulation. Due to the non-specificity of the drug–cyclodextrin interactions, incorporation of new molecules such as polysaccharides into the bulk may produce interference, competition or promotion of the formation of the inclusion complex [18–19]. Therefore, it became necessary to study the effect of these polysaccharides on the solubility of the fluconazole–HPBCD or –SBECD complex. As can be observed in Figure 2, the addition of gellan gum or κ-carrageenan at a percentage of 0.5% into the cyclodextrin solutions does not modify the fluconazole solubility as compared with the cyclodextrins alone (one-way ANOVA, α n.s).

### Cell toxicity and ocular safety of cyclodextrins

Cyclodextrins have been proposed as an excipient to formulate a variety of lipophilic drugs into eye drops. CDs are known to facilitate the preparation of drugs in eye drop formulations that otherwise might not be available for topical use by improving their absorption and stability and decreasing local irritation [20].

Considering that corneal stroma plays a key role in a wide range of ophthalmic disease frames, substantial areas of research are currently focused on achieving non-toxic concentrations of ocular products able to keep the tissue intact [21]. In this regard, large hydrophilic cyclodextrins such as HPBCD and SBECD have been considered as safe materials for the formulation of aqueous eye drop solutions since they do not cross the lipophilic cornea. Based on previous toxicological eye studies [22], in the current work, we propose the comparison of two of the most relevant cyclodextrins by using a new methodology, real-time xCELLigence, based on live stromal cell analysis, which evaluates dynamic live cell monitoring [23].

**Figure 3:**
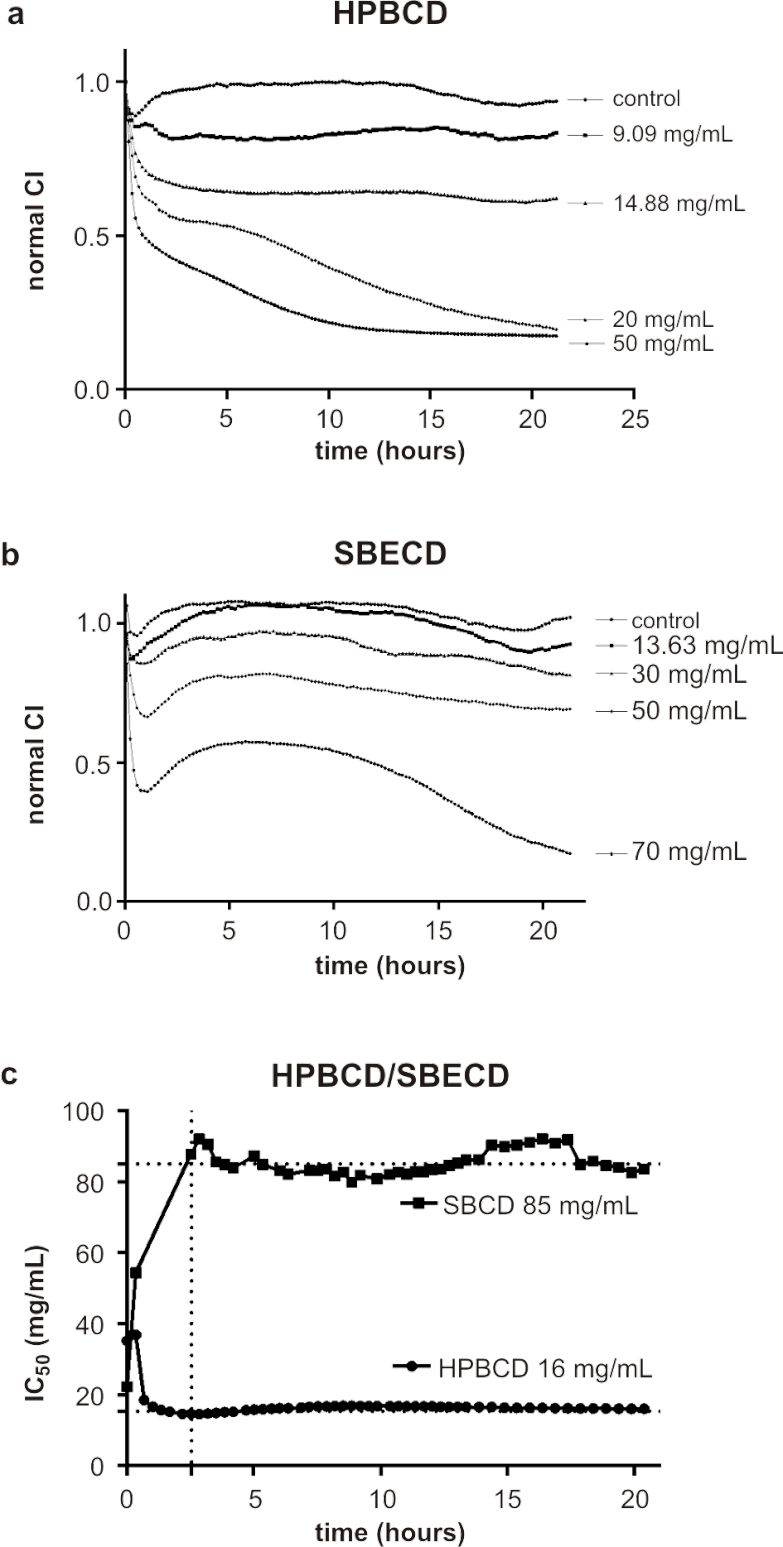
Ocular cytotoxicity studies of HPBCD and SBECD in primary corneal human keratocytes, using real-time xCELLigence impedance analysis. (a) Kinetic curve survival rates for HPBCD; (b) Kinetic curve survival rates for SBECD; (c) Evolution of IC_50_ vs time for HPBCD and SBECD. IC_50_ values at each time were calculated using (a) and (b); IC_50_ represents the concentration that causes a reduction in the survival of 50% of the cells.

The kinetic curve of cell survival rates obtained using real-time xCELLigence impedance analysis is shown in Figure 3. Both cyclodextrin derivatives induced a gradual decline in the cell survival rate over a 20 hour exposure period, suggesting that CDs may cause some corneal cell toxicity. Further analysis of the cell survival rate kinetic curves shows that these effects were time and dose dependent. According to the dynamic cell response to serial doses of CDs shown in Figure 3a for HPBCD and Figure 3b for SBECD, the real time method allowed the calculation of the corresponding IC_50_ value (i.e., the concentration that causes a reduction in the survival of 50% of the cells) at each time point. The representation of the time-dependent IC_50_ curve (Figure 3c) allowed the rate of the toxic effect to be determined and also the detection of other possible effects during the measurement.

As shown in Figure 3c, HPBCD induces cytotoxicity immediately after its administration, reaching an initial IC_50_ value of 40 mg/mL. Subsequently, its value decreases quickly during the exposure period and reaches a stable value of 16 mg/mL after 2.5 hours, which is maintained for over 15 hours. This result shows that the cytotoxicity of HPBCD is enhanced with increasing exposure time.

In the case of SBECD, cytotoxicity was observed immediately after its addition, reaching an initial IC_50_ of 22 mg/mL, which is slightly lower than the initial data observed for HPBCD. It then increases gradually during the exposure period and reaches a stable value of 85 mg/mL at 2.5 hours and remained stable over the next 15 hours. Thus, in contrast to HPBCD, SBECD shows higher initial cytotoxicity that decreases during the exposure time.

One of the advantages of the time-dependent IC_50_ curve analysis over the end-point measurement is that it permits understanding the dynamic toxicity behavior, and helps to identify the range of exposure time, which is interesting to investigate in accordance with the mode and method of administration [24].

Upon administration of topical eye formulations the drainage away from eye surface is essentially completed at around 90 s. Under these conditions, the ocular surface is in contact with high drug concentrations only for very short periods of time. For this reason, knowing the initial cytotoxicity is crucial. Bioadhesive formulations as ion-sensitive gels have a high retention rate in the eye; however, the contact time between the ingredients and the surface rarely exceeds a few hours. Therefore, it is critical to understand the cytotoxic effects which occur over short contact periods.

One important aspect is that under normal conditions, the eye can accommodate only a very small volume of solution [25], which will undergo a significant and rapid dilution. Thus, the concentration of cyclodextrins on the ocular surface after its administration will be significantly lower with respect to that which can produce cytotoxicity.

**Figure 4:**
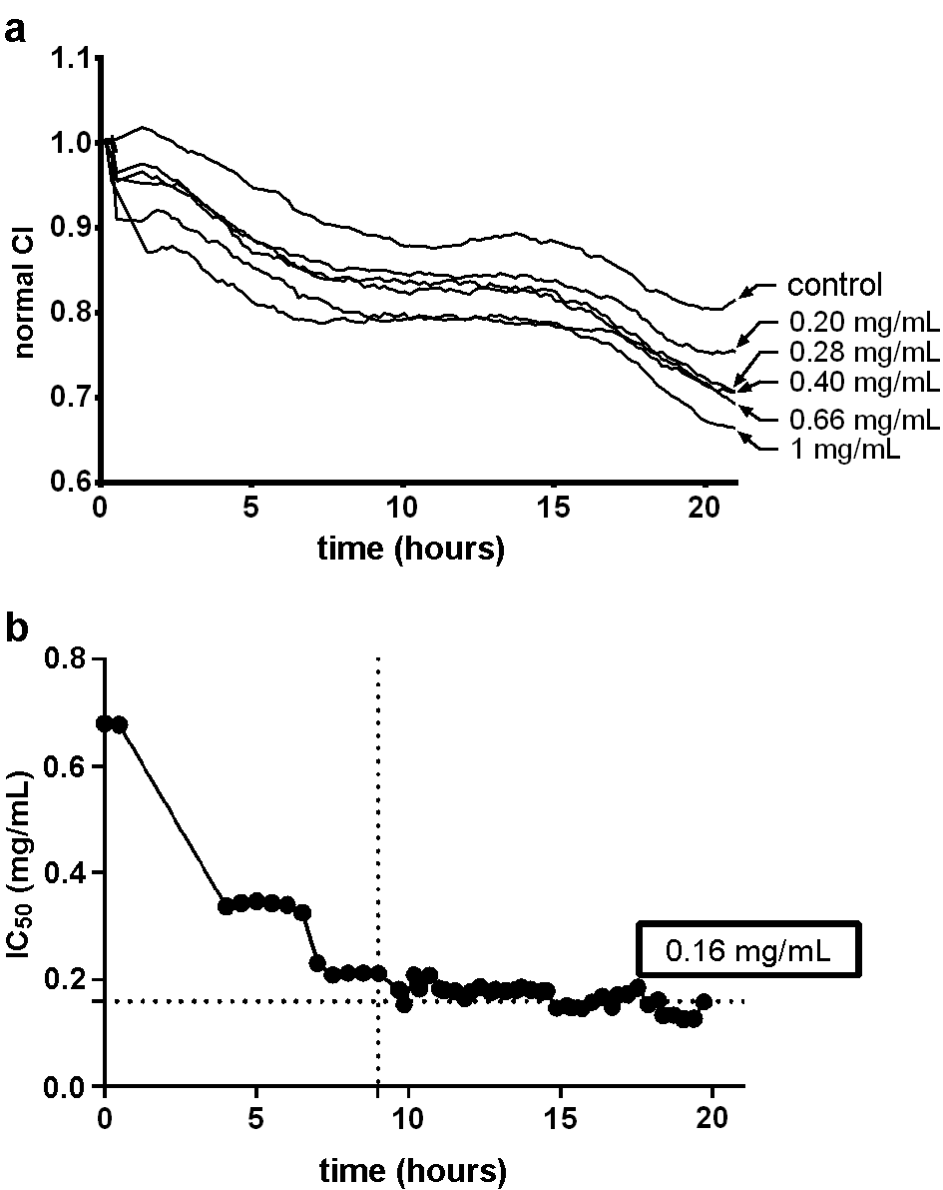
Ocular cytotoxicity studies of fluconazole in primary corneal human keratocytes obtained using real-time xCELLigence impedance analysis. (a) Kinetic curve survival rates; (b) evolution of IC_50_ vs time.

In Figure 4 the kinetic survival rate curves and the temporal evolution of the IC_50_ of fluconazole are shown. The results indicate that the cytotoxicity is time and dose dependent: an increase in these parameters results in an increase in the cytotoxicity. Fluconazole shows an initial IC_50_ of 0.69 mg/mL that decreases until it reaches an equilibrium value of 0.16 mg/mL after 9 hours of contact. Hence, this drug has a higher cytotoxicity than HPBCD or SBECD.

**Figure 5:**
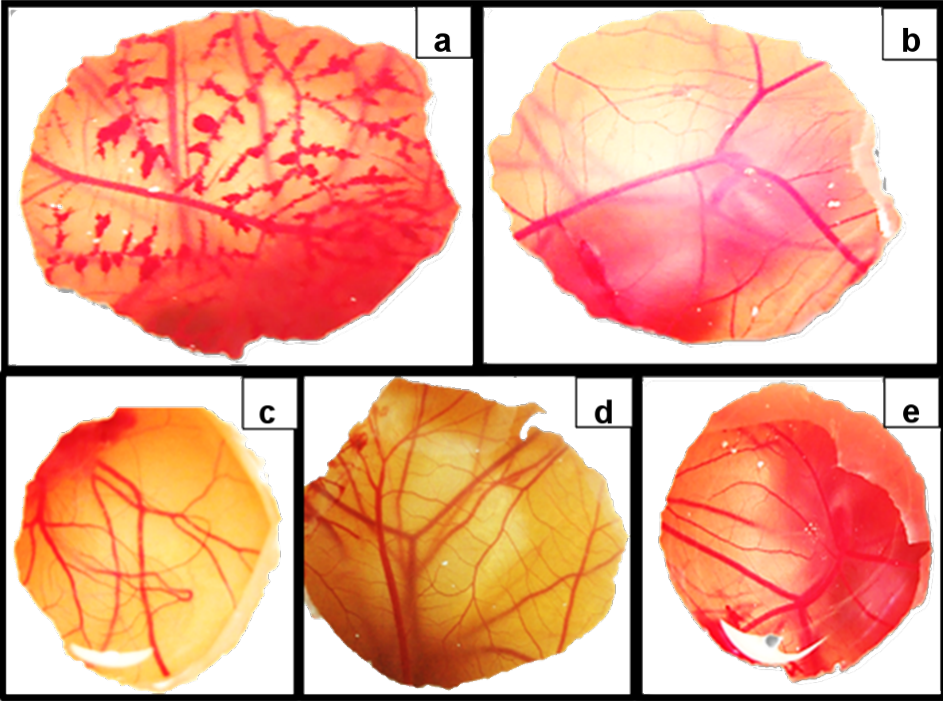
HET-CAM assay: (a) positive Control (NaOH 0.1 N); (b) negative control (NaCl 0.9%); (c) HPBCD 10 mg/mL; (d) SBECD 10 mg/mL; (e) fluconazole (2 mg/mL). Photographs show the status of the vessel at the end of the experiment.

To complement the cytotoxicity studies, the Hen's Egg Test method (HET-CAM) was used to quantify potential irritation [26]. In this assay, hemorrhage, lysis or coagulation in the blood vessels of the chorioallantoic membrane (CAM) was not observed. CDs, fluconazole and final gel formulations (0.65%, 0.75% and 0.82% gel with and without HPBCD) showed no irritation based on the obtained irritation score (IS) of zero for all of these compounds (Figure 5). Therefore, the gels and their components are considered suitable for ocular use from an irritation study point of view.

### Ion-sensitive ophthalmic gel

Because both cyclodextrin derivatives were shown to be safe, non-irritant and able to form complexes with fluconazole (with similar stability properties), both substances were considered as good candidates for the development of hydrogels. In this work, we have chosen HPBCD to solubilize fluconazole because although it showed comparable cytotoxicity effects compared to SBECD over short exposure periods, it has a lower molecular weight, and therefore, higher drug solubilization capacity per gram of CD, allowing for gels with lower proportions.

Ion-sensitive hydrogels synthesized without HPBCDs incorporated a concentration of fluconazole of 0.2% which is limited by its low water solubility. Hence, the incorporation of HPBCDs allows for hydrogels with higher doses. In the present study, we have selected the 1% concentration which requires the incorporation of 74.25 mg of HPBCD.

Bioadhesion is an interfacial phenomenon occurring between a polymer and a biological surface. Due to the complex nature of polymers and molecules present in biological surfaces, many factors determine the strength and duration of the adhesion. However, the specific interactions in the polymer/biological substrate interface are governed by both the properties of the polymer and the nature of the substrate [27].

**Figure 6:**
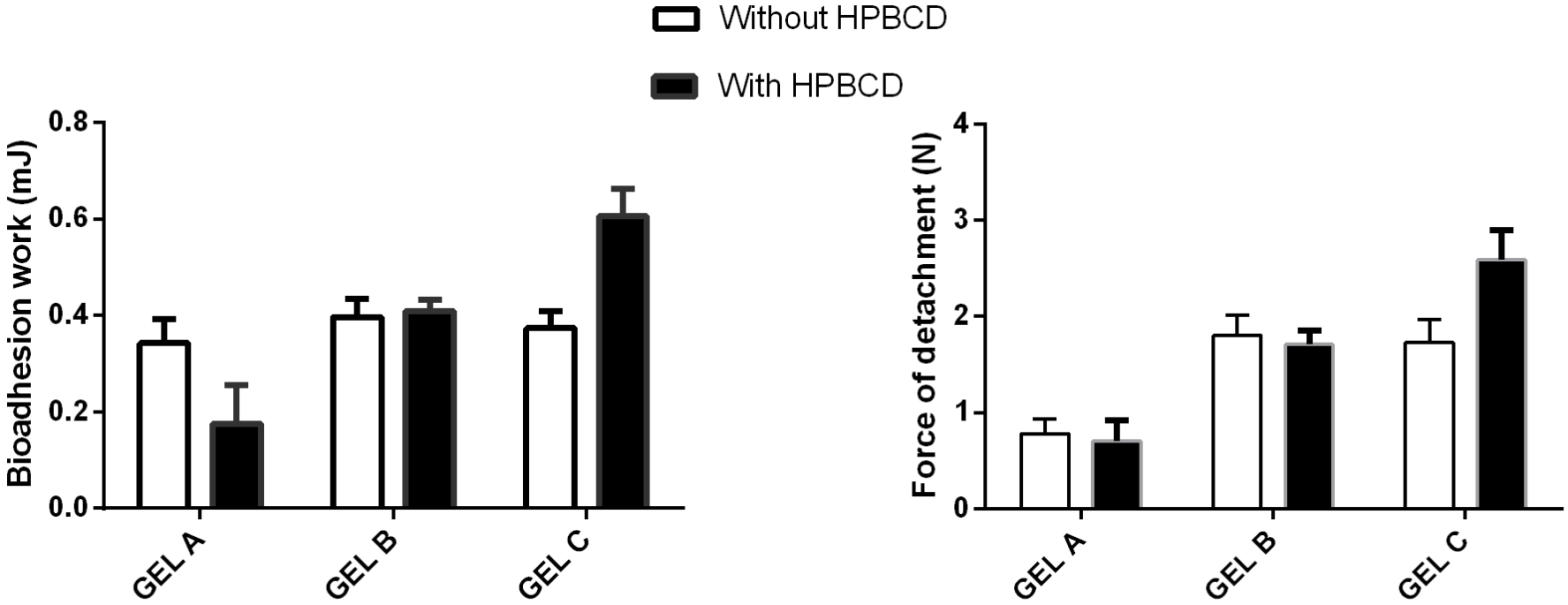
Bioadhesive properties (bioadhesion work and maximum force of detachment) of ion-sensitive hydrogels using tanned leather as a substrate (mean ± SD, *n* = 6).

Figure 6 shows the bioadhesive behavior of the ion-sensitive gels in tanned leather in the presence of simulated tear fluid, and the effect of the HPBCDs on the bioadhesive work and detachment force. In absence of HPBCDs, no significant influence on the concentration or proportions of polysaccharides in the gels was observed and only differences in the maximum force detachment were observed for the gel (α < 0.05).

Although the incorporation of HPBCDs does not induce significant changes in the bioadhesive behavior, a decrease in the bioadhesive work of gel A (α < 0.05) and an increase in both bioadhesive parameters for gel C was observed (α < 0.05).

**Figure 7:**
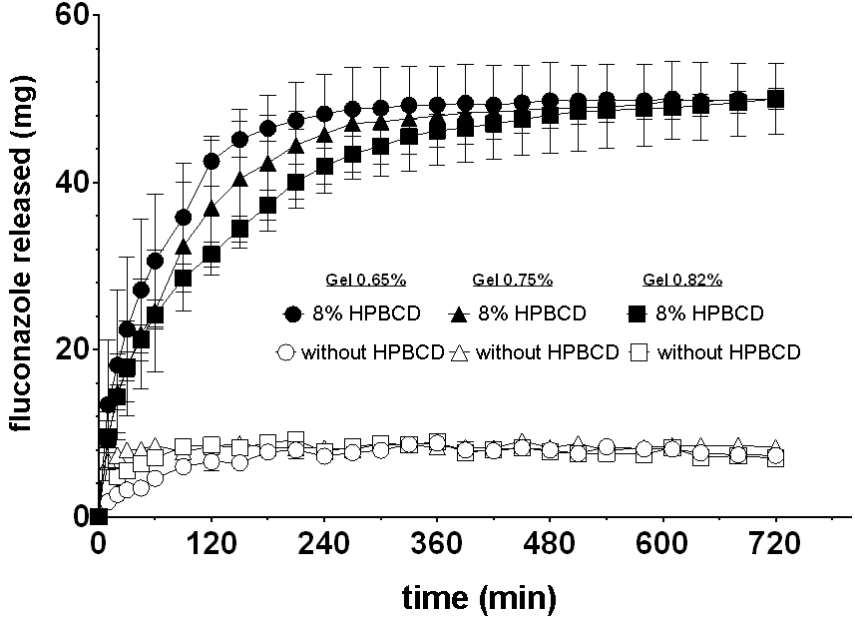
Release profiles of fluconazole in simulated tear fluid from the gels with (closed symbols) or without (open symbols) HPBCDs and different proportions of polysaccharides (mean ± SEM, *n* = 3).

The release of fluconazole from the ion-sensitive hydrogels containing the free and complexed drug is shown in Figure 7. All compositions tested underwent rapid gelation upon immersion in simulated tear fluid. A rapid sol–gel transition was induced by the interaction of gellam gum and κ-carrageenan with the monovalent and divalent ions of the simulated tear fluid. This gelation promotes controlled drug release, suppressing their premature release.

The release of fluconazole from ion-sensitive gels is performed relatively fast during the first hour, being completely released by the end of this period. However, gels made with cyclodextrins prolong the drug release for approximately 4 hours. Additionally, a higher dose of fluconazole incorporated in the hydrogels containing cyclodextrins allowed the release of a higher quantity of the antifungal (α < 0.05). Insignificant differences in the drug release properties were observed as a function of the polysaccharide concentration or in the ratio of gels with and without cyclodextrins.

Table 2 shows the results of the fitting to determine the quantity of fluconazole release up to 120 min using Higuchi kinetics [28]. The good fits obtained suggest that release is controlled by the diffusion of the drug across the hydrogels. This mechanism is consistent with the observed differences in the release rate due to the presence of cyclodextrin. Gels incorporating HPBCDs showed higher release rates which remained stable. Despite the fact that the formation of the inclusion complex represents only a incremental increase in the molecular volume as compared with the drug alone (decreasing their diffusivity), the significant increase in the fluconazole concentration in the gel results in an increase of the concentration gradient between gel and release medium, providing a fast and more prolonged drug release.

**Table 2:** Fitting results yielding the quantity of fluconazole release up to 120 min using the Higuchi release kinetics.^a^

	With HPBCD		Without HPBCD	
	*k* (mg × min^−0.5^) Mean ± sem	R^2^	*k* (mg × min^−0.5^) Mean ± sem	R^2^

Gel A	2.92 ± 0.10	0.9924	0.61 ± 0.03	0.9873
Gel B	3.81 ± 0.08	0.9972	0.79 ± 0.06	0.9676
Gel C	3.41 ± 0.08	0.9968	0.64 ± 0.20	0.6235

^a^Higuchi release kinetics 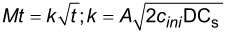 [21], were *A* is the area, *c*_ini_ the initial drug concentration in the gel, D is the diffusivity and *C*_s_ the drug solubility.

These results agree with those published by other authors [29–30], which further demonstrates that the incorporation of cyclodextrins in gel formulations can delay the release of the drug over time.

## Conclusion

In this study an ophthalmic drug delivery system was developed using mixtures of an in situ-gelled vehicle and CDs containing high doses of fluconazole. An ophthalmic gel containing HPBCDs was demonstrated to be safe and bioadhesive. In the presence of simulated tear solution, the in situ-gelled vehicle was able to form a strong gel following the phase transition that allows controlled drug release. Finally, the inclusion of HPBCDs in the in situ-formed gel allows for more effective control and a significant increase in the fluconazole release.

## Experimental

### Materials

In this study, the following substances were used: Sulfobutyl ether-β-cyclodextrin (SBECD, Captisol^®^, average degree of substitution for the sulfobutyl group: 6.6, average MW 2179) was a generous gift from Cydex Inc. (Lenexa, KS); 2-hydroxypropyl-β-cyclodextrin (HPBCD, Kleptose HPBCD^®^ with a molar substitution of 0.65 and MW 1399 Da) was a generous gift from Roquette-Laisa (España); fluconazole (FC) was purchased from ACOFARMA (España); gellam gum (GG, Kelcogel CG-LA, CPKelco); and κ-carrageenan (CK, Genugel^®^ carrageenan CG-130, CPKelco).

#### Phase solubility diagram

Drug–cyclodextrin interactions can be studied by a variety of techniques, most of which examine the behavior of mixtures of the cyclodextrin (host) and drug species (guest) in solution. A more accurate method for the determination of the efficiency of the cyclodextrin solubility is to determine their complexation efficiency (CE) [31], that is, the concentration ratio between cyclodextrin in a complex and free cyclodextrin. The CE is calculated from the slope of the phase solubility diagram, and is independent of both S_0_ and S_0,extrap_, and thus more reliable when the influences of polymers (which are contained in an ophthalmic gel) on the solubility are being investigated.

A phase solubility diagram technique was employed to estimate the stability constants of fluconazole, HPBCD and SBECD. Solubility measurements were carried out according to the method of Higuchi and Connors [14] and by following the protocols previously described by Anguiano-Igea et al. [15]. Excess amounts of fluconazole were added to a series of aqueous solutions of increasing concentrations of cyclodextrin. These solutions were shaken in an orbital shaking bath (VWR) at 25 °C and 70 rpm for 7 days in order to reach equilibrium.

After equilibrium was attained, an aliquot was centrifuged for 0.5 h at 12,500 rpm (SIGMA 2-16P) and 1 mL aliquots of the supernatant were diluted 100 times. The concentration of fluconazole in each sample was determined using a spectrophotometer diode array (Hewlett Packard 8452A, λ = 260 nm). The final values presented for the fluconazole solubility measurements are the means of three replicate measurements.

The phase solubility diagrams were obtained by plotting the mean solubility against the cyclodextrin concentration. The apparent stability constant, assuming the formation of a cyclodextrin inclusion complex with a 1:1 stoichiometry (*K*_1:1_), was calculated from the slope and the drug solubility (S_0_) or the intercept (S_0,extrap_) from linear regions obtained by least squares regression using the following equation:

[1]
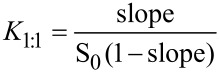


The parameter complexation efficiency CE is given by:

[2]
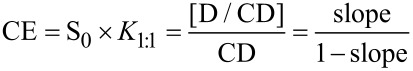


In addition, the D:CD ratio can be calculated using the CE according to:

[3]
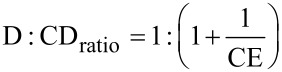


In order to study the influence of the presence of the two polysaccharides on the complex solubility, the drug solubility in an aqueous solution of gellam gum or κ-carrageenan at 0.5% and HPBCD or SBECD at 5% was determined by the same methodology described above.

#### Cell toxicity and ocular safety of CDs

To determinate the ocular toxicity of the cyclodextrins used in the gels, both varieties were tested in primary corneal human keratocytes (HCK) and Hen's Egg Test.

#### Isolation of human keratocytes

The study was conducted according to our institution’s guidelines and the declaration of Helsinki. To isolate human keratocytes, a modification of the method proposed by Ramke et al was used [32].

#### Cell cytotoxicity assay

Cell cytotoxicity was assessed by using the label-free xCELLigence system (ACEA Biosciences, San Diego, CA) which allows for real-time monitoring. Using this platform, the cell index (CI) was the parameter used to represent the cell status based on the measured electrical impedance [23,33].

Briefly, 3000 cells/well (E-plates, 16 wells) were seeded and incubated for 24 hours until the CI reached a range of 1.0–1.2, indicating about 60% cell confluence. At that point, the cell culture medium was aspirated to perform cell treatment with the CDs diluted in culture medium of different concentrations. The data obtained was represented in dose response curves.

The IC_50_ values represent the concentration of CDs producing a 50% reduction of CI and the concentration of CDs producing a 50% reduction as compared to the negative control (culture medium). The IC_50_ values were obtained directly from the dose response curves, which has been previously described [34].

#### The Hen's Egg Test (HET)

The HET-chorioallantoic membrane (HET-CAM) is used to quantify the potential membrane irritation, providing an alternative to the Draize methodology [35]. Briefly, freshly fertilised white leghorn eggs, with a weight of 50–60 g, were incubated at 37.5 °C and a relative humidity of 62 ± 7.5% for 9 days in an incubator with an automatic rotating device. After removing the egg shell covering the air cell with an electric drill and cutting through the inner egg membranes, 0.3 mL of test substance was applied (fluconazole: 2 mg/mL, CDs: 10 mg/mL and final gel formulations: 0.65%, 0.75% and 0.82% gel with and without HPBCD) was applied onto the vasculated chorioallantoic membranes (CAM) of at least three eggs. The CAM, the blood vessels (including the capillary system), and the albumen were all observed over time (300 s) under a stereomicroscope (Olympus SZ-STN) and scored for effects (hemorrhage, coagulation and partial lysis). Sodium hydroxide 0.1 N served as positive control and sodium chloride 0.9% as a negative control. Both cyclodextrins were placed in the CAM for determination of irritation score (IS) by the methodology based on described in protocol No. 96 from INVITTOX [26].

#### Preparation of ophthalmic gels

Ion-sensitive hydrogels were prepared using three different concentrations of polysaccharides. Gel A was prepared using a 0.82% (w/v) of a mixture of GG and CK in 4:1 ratio; Gel B using 0.75% (w/v) of a mixture of GG and CK in ratio 2:1, and Gel C using a 0.65% (w/v) of a mixture of GG and CK in ratio 1:1. The concentrations and ratios of the polymer were selected in function of a previous work (unpublished data). The solutions were prepared by dispersing in warm distilled water (55 °C) and stirring for 24 hours.

#### Incorporation of drug and CD–drug complexes into ophthalmic gels

Fluconazole was incorporate at the gel at a concentration of 2 mg/mL by dispersion of the solid drug into the gel under magnetic stirring. To incorporate the fluconazole–CD complex, cyclodextrin were previously dissolved in distilled water. Next fluconazole (10 mg/mL) was dissolved in the CD solution. Finally the polymers were incorporates in the different concentrations and ratios to obtain the gels.

#### Characterization of bioadhesion

Bioadhesive capacity was determined by measuring the maximum detachment force and the bioadhesion work employing a TA-XT Plus texture analyzer (TA Instruments, Newcastle, UK) using tanned leather as substrate. The substrate was selected because tanned leather is a good model for determining bioadhesion [36]. Tanned leather cylinders of 2 cm diameter were adhered on the upper and the lower support by using double layer paper and 0.2 mL of the sample was deposited on the lower support. Then a compression/extension stage using compression and extension rates of 1 mm/s (maintaining an applied contact force of 0.5 N over 300 s) was applied. Force versus elongation was recorded and maximum force and bioadhesion work was calculated.

#### In vitro release of fluconazole from ophthalmic ion-sensitive gel

The in vitro release of fluconazole–CDs from the in situ-gelled systems was studied using a membraneless model using an no. 2 USP automated dissolution testing apparatus consisting of a Prolabo Dissolutest fitted with a Hewlett Packard 8452A diode array spectrophotometer. Similar membraneless methods using a modified dissolution testing apparatus and high volumes of simulated tear fluid was implemented for another study in order to characterize in vitro drug release of ophthalmic gels [37–38]. These models were demonstrated as more useful than membrane models (i.e., using Franz cells) as they mimic the clearance effect and dilution of the tear in the eye surface.

Hydrogels (5 g) were placed in open, 4 cm diameter, glass containers which were placed in the bottom of a beaker filled with 400 mL of simulated tear fluid at 37 °C and 75 rpm. The in situ gel formation occurred during the sol–gel transition upon contact with the simulated tear fluid.

#### Statistical analysis

The statistical analysis was made using the software, Statgraphics Centurion XVI (Stat Point Technologies, Inc.). The solubility analysis was performed using a one-way ANOVA analysis and the Student–Newman–Keuls method as multiple comparison tests. The bioadhesion work and maximum detachment force were analyzed by a two-way ANOVA analysis using the concentration of polymers and the presence of CDs as independent factors and the Student–Newman–Keuls method was used as a multiple comparison test. Finally, release study analysis was developed by applying a two-way ANOVA to the volume of fluconazole released using time and formulation as independent factors. Again, the Student–Newman–Keuls method was used as a multiple comparison test.

## References

[1] Miller D (2013). Expert Opin Pharmacother.

[2] Keay L J, Gower E W, Iovieno A, Oechsler R A, Alfonso E C, Matoba A, Colby K, Tuli S S, Hammersmith K, Cavanagh D (2011). Ophthalmology.

[3] Mravii I, Dekaris I, Gabri N, Romac I, Glavota V, Mlinari E, Srinivasan M (2012). Keratitis.

[4] Kathiravan M K, Salake A B, Chothe A S, Dudhe P B, Watode R P, Mukta M S, Gadhwe S (2012). Bioorg Med Chem.

[5] Pfaller M A, Diekema D J, Sheehan D J (2006). Clin Microbiol Rev.

[6] Gratieri T, Gelfuso G M, de Freitas O, Rocha E M, Lopez R F V (2011). Eur J Pharm Biopharm.

[7] Pawar P, Kashyap H, Malhotra S, Sindhu R (2013). BioMed Res Int.

[8] Achouri D, Alhanout K, Piccerelle P, Andrieu V (2013). Drug Dev Ind Pharm.

[9] Kushwaha S K S, Saxena P, Rai A K (2012). Int J Pharm Invest.

[10] Liu Z, Li J, Nie S, Liu H, Ding P, Pan W (2006). Int J Pharm.

[11] Chen X, Li W, Zhong W, Lu Y, Yu T (1997). J Appl Polym Sci.

[12] Coviello T, Matricardi P, Marianecci C, Alhaique F (2007). J Controlled Release.

[13] Otero-Espinar F J, Blanco-Méndez J (2014). Curr Top Med Chem.

[14] Higuchi T, Connors K A (1965). Phase-Solubility Techniques. Advances in Analytical Chemistry and Instrumentation.

[15] Anguiano-Igea S, Otero-Espinar F J, Vila-Jato J L, Blanco-Méndez J (1997). Eur J Pharm Sci.

[16] Upadhyay S K, Kumar G (2009). Chem Cent J.

[17] Kutyła M J, Lambert L K, Davies N M, McGeary R P, Shaw P N, Ross B P (2013). Int J Pharm.

[18] Nogueiras-Nieto L, Alvarez-Lorenzo C, Sandez-Macho I, Concheiro A, Otero-Espinar F J (2009). J Phys Chem B.

[19] Jansook P, Loftsson T (2009). Int J Pharm.

[20] Loftsson T, Stefánsson E (2002). Acta Ophthalmol Scand.

[21] Kilic C, Girotti A, Rodriguez-Cabello J C, Hasirci V (2014). Biomater Sci.

[22] Niles A L, Moravec R A, Riss T L (2008). Expert Opin Drug Discovery.

[23] Xing J Z, Zhu L, Gabos S, Xie L (2006). Toxicol In Vitro.

[24] Chen H, Cui L, Jiang X-Y, Pang Y-Q, Tang G-L, Hou H-W, Jiang J-H, Hu Q-Y (2012). Food Chem Toxicol.

[25] Kumar S, Karki R, Meena M, Prakash T, Rajeswari T, Goli D (2011). J Adv Pharm Technol Res.

[26] (2014). Hen’s Egg Test on the Chorioallantoic Membrane (HET-CAM) INVITTOX no. 96.

[27] Vallejo Diaz B M, Perilla J E (2008). Rev Colomb Cienc Quim-Farm.

[28] Siepmann J, Peppas N A (2011). Int J Pharm.

[29] Otero Espinar F J, Torres-Labandeira J J, Alvarez-Lorenzo C, Blanco-Méndez J (2010). J Drug Delivery Sci Technol.

[30] Bibby D C, Davies N M, Tucker I G (2000). Int J Pharm.

[31] Loftsson T, Hreinsdóttir D, Másson M (2005). Int J Pharm.

[32] Ramke M, Lam E, Meyer M, Knipper A, Heim A (2013). Mol Vision.

[33] Xing J Z, Zhu L, Jackson J A, Gabos S, Sun X-J, Wang X-b, Xu X (2005). Chem Res Toxicol.

[34] Ceriotti L, Ponti J, Broggi F, Kob A, Drechsler S, Thedinga E, Colpo P, Sabbioni E, Ehret R, Rossi F (2007). Sens Actuators, B.

[35] Fernández-Ferreiro A, González Barcia M, Gil Martínez M, Blanco Mendez J, Lamas Díaz M J, Otero Espinar F J (2014). Farm Hosp.

[36] Blanco-Fuente H, Anguiano-Igea S, Otero-Espinar F J, Blanco-Méndez J (1996). Int J Pharm.

[37] Lin H-R, Sung K C (2000). J Controlled Release.

[38] Wu H, Liu Z, Peng J, Li L, Li N, Li J, Pan H (2011). Int J Pharm.

